# The Cultural Art Aesthetic Behavior of Entrepreneurship Education for College Students in the Characteristics of Film and Television Media

**DOI:** 10.3389/fpsyg.2022.880649

**Published:** 2022-07-04

**Authors:** Wei Sun, Hongkai Wang, Li Wang, Lele Ye, Peiyao Chen

**Affiliations:** ^1^Zhujiang College, South China Agricultural University, Guangzhou, China; ^2^School of Media Communication, Shenzhen University, Shenzhen, China; ^3^College of Journalism and Communications, Shih Hsin University, Taipei City, China; ^4^New Media Animation Department, Jilin Animation Institute, Changchun, China; ^5^Zhijiang College, Zhejiang University of Technology, Shaoxing, China; ^6^School of History and Culture, University of Birmingham, Birmingham, United Kingdom

**Keywords:** new media age, film and TV media characteristics, college Students’ cultural aesthetic education, impact and strategy, cultural art aesthetic behavior, entrepreneurship education

## Abstract

This exploration aims to promote the organic integration of innovation and entrepreneurship education and art education, further promote the reform of college Students’ cultural and aesthetic education, improve college Students’ aesthetic perception ability, and help contemporary colleges establish a correct political morality. This thesis aims to further promote the reform of college Students’ cultural and aesthetic education, improve college Students’ aesthetic perception ability, and help contemporary colleges establish correct political and moral values. First, the connotation of college Students’ aesthetic education and the definition of cultural aesthetics are introduced, which is based on the characteristics of two-way interaction, multiple selectivity, timeliness and popularization of film and television media in the new media era; then, the way of questionnaire is adopted. With five universities as the research object, 250 questionnaires are distributed, and 235 valid questionnaires are collected, with a valid response rate of 94%. Finally, through the six questions, it is concluded that 68.9% of the students watch 3–5 h a day, and 4.3% of the students watch more than 7 h; 89.4% of the students hold that the same products as stars in film and television will exert an impact on consumption. Film and television culture and art have a positive and negative impact on college Students’ cultural aesthetic perception. The positive impact is that the film and television media not only provides a good way to cultivate the aesthetic perception ability of contemporary college students, but also helps them to establish the correct aesthetic values. The negative impact is mainly reflected in two levels, namely, the vulgarization of film and television media works and the consumption of aesthetic concepts. The advantage of this exploration is to put forward the reform measures of college Students’ cultural and artistic aesthetic education under the current educational background in China to help colleges better carry out college Students’ cultural and artistic aesthetic education. Based on this, the reform measures of college Students’ cultural aesthetic education under the current education in China were put forward, so as to help colleges and universities better carry out college Students’ cultural aesthetic education.

## Introduction

With the development of science and technology and the arrival of the new media era, the film and television media show different characteristics. Traditional film and television is often a process of single-direction transmission, and the audience is more passive to accept the culture transmitted by the film (Szu et al., 2017; Ophir et al., 2020). Under the influence of new media technology, film and television media have two-way interaction. Specifically, students play the role of film critics and aesthetic culture leaders. Now, most video sites or mobile terminals have increased the function of barrage. The appearance of barrage has made the private complained become a part of watching culture, making people watching the movie and TV works and being influenced by the barrage culture (Druick, 2016).

In the era of new media, film and television media also have diversification and selectivity (Shen et al., 2019). Generally speaking, mainstream culture is the core of social and cultural communication and the cornerstone of the development of national culture. In the early stage of the development of network technology, the content of film and television culture is relatively controllable. However, with the rapid development of the Internet, a large number of foreign programs are flooded in the major video websites. On the contrary, in China, local film and television culture has been greatly marginalized impact (Duffy, 2016; Gill, 2016). Taking American film and television factories as an example, their advanced equipment, professional practitioners, and skilled narrative skills make Chinese college students more likely to choose such films and television to spend their leisure time. The cultural art aesthetic instillation of American personal heroism has become an inevitable trend of movie watching. But this kind of non-tidal current cannot be completely avoided by means of coercion. Film and television media art is a unique artistic form of expression. It is also known as the most influential, energetic and vital way of cultural and artistic education in the twenty-first century (McNicholas Smith and Tyler, 2017). Its dynamic visual art features are incomparable to other visual arts, but it can well integrate the advantages of other visual arts. In addition, film and television media art not only has the advantages of visual art, but also has the advantages of other forms of art, and more infectious and impact than other forms of art (Mühling et al., 2017). For example, comparing the film and television media art with the visual art stage play and the concert of the auditory art, the performance forms concentrate their characteristics, which have more artistic expression and vitality. Secondly, compared with the literature of the art of thinking and imagination, it is more impressive and influential in terms of expression (Twenge et al., 2017).

In addition, cultural art aesthetics is a concrete manifestation of human characteristics in aesthetic activities. It is not only a dynamic process of artificial creation, but also a manifestation of material civilization (Buckingham, 2015). For the education of contemporary college Students’ aesthetic culture, it is proved that integrating aesthetic consciousness into college Students’ thoughts can form artistic expression of beauty, thus cultivating college Students’ aesthetic interest and cultural taste, deepening their pursuit of truth, goodness and beauty, and establishing correct values, outlook on life, and world outlook (Lee et al., 2016). Aesthetic education is an important part of comprehensive quality education for contemporary college students, and also an important part of ideological and political education for college students. Aesthetic education has its unique function and significance. Its goal is to cultivate and develop college Students’ sensibility, including sensibility, appreciation, creativity, and imagination, so as to cultivate their sound and noble personality and shape perfect personality, thus realizing the ultimate pursuit of harmonious unity of human and nature, human and society, and human and human’s own sensibility and rationality (Trinder, 2016; Gutiérrez-Martín and Torrego-González, 2018). The aesthetics of college students is closely related to the mainstream output of society, and they affect each other. Perfecting college Students’ aesthetic concept is an effective form to promote college Students’ personality development, which is related to the practice of socialist core values. The quality of a person, a society, and a country depends on its cultural connotation. In other words, aesthetic ability and artistic quality are the key to judge the realm of individual, society, or country. Art can achieve a perfect person and a perfect country. Therefore, the so-called “cultural power” is “artistic/aesthetic power.” In order to “establish” and “strengthen” the cultural power, it is necessary to vigorously popularize national art education and improve the aesthetic ability of the whole people. The most effective way to improve national artistic quality is to integrate art professional education with innovation and entrepreneurship education through art education. Art education can improve the national artistic taste and artistic aesthetic ability, constantly improve their own life taste, and then make them pursue higher beauty and character to improve their way of life, work and dealing with people, and even all aspects of social life. Finally, it helps achieving the highest level of “educating people through culture,” meeting the world challenge of cultural competition, and realizing the Chinese dream of becoming a cultural power of the Chinese nation (Chen, 2019).

In modern China, where innovation and entrepreneurship education has become the focus of teaching reform in colleges, how to organically integrate innovation and entrepreneurship education with art education is a key problem at present (Feng and Chen, 2020). In 2020, the sudden outbreak of COVID-19 had a profound impact on all walks of life in the world and also deeply affected the education of innovation and entrepreneurship for college students. With the rapid development of the digital economy in the post epidemic era, the Internet working mode has become a new business form, which puts forward new requirements for college Students’ innovation and entrepreneurship ability. In the post epidemic era, the rapid development of new business forms of the digital economy has accelerated the deep integration of Internet platforms and entrepreneurial units. Innovation and entrepreneurship education is a process of cultivating Students’ objective understanding of problems, in-depth analysis of problems and creative problem-solving, while aesthetic education is a process of cultivating Students’ aesthetic ability and quality of perceiving man and nature, man and society, man and heart (Deng et al., 2021). The two are highly consistent. With the continuous development and progress of the times, the advantages of film and television media art and its educational role have been widely concerned by educators in China and foreign countries. Moreover, many colleges and universities have opened corresponding film and television media courses, implemented modern education in film and television communication, and also made the advantages of film and television media art more prominent. Therefore, the study of film and television media is of great practical significance for the improvement of college Students’ aesthetic value and the formation of their personal moral qualities and accomplishments. Questionnaire is adopted to study the characteristics of film and television media and college Students’ cultural and art aesthetic education in the new media era. The research innovation is that the reform measures of college Students’ cultural and art aesthetic education under China’s current education are put forward, so as to help colleges better carry out college Students’ cultural and art aesthetic education. This exploration is structured as follows. First, it expounds the strategy of the combination of film and television media and college Students’ culture, art, and aesthetic education. Next, the influence of film and television culture on college Students’ values and the value of college Students’ cultural aesthetic education under the background of innovation and entrepreneurship education are analyzed. Finally, through a questionnaire, the positive and negative effects of film and television culture and art on college Students’ cultural aesthetic perception are studied.

## Materials and Methods

### Strategies of Combining Film and Television Media With College Students’ Cultural Art Aesthetic Education

Before analyzing the application strategy, five characteristics of the film and television media are elaborated.

#### Instantaneity

The so-called instantaneity refers to the current situation that the film and television media express and reflect. It can satisfy the psychological characteristics of the audience in hunting for the latest social trends to the greatest extent. Especially some TV programs, such as sports competitions, literary performances, celebration speeches, etc., they often use live broadcasting mode, which brings great ornamental charm and psychological stimulation to the audience (Kanellopoulou et al., 2019). This instant performance enables all ethnic groups to experience and obtain the charm and excitement of the ongoing events and situations at a common moment. The Spring Festival Gala is one of the most representative examples (Bridgstock and Cunningham, 2016).

#### Popularity

Popularity refers to the spread scope of the current film and television media. An uneducated person may not read books and newspapers, but he can see movies and TV works (Banks and Oakley, 2016). This proves to a certain extent the popularity of the film and television media, and its content can be understood and perceived by the public. In this sense, the film and television media have the connotative characteristics of both elegant and popular appreciation (Fedorov and Levitskaya, 2016).

#### Intuitiveness

Intuitiveness means that the film and television media works are presented through dynamic forms such as sound and image. Generally speaking, film and television works are all presented through sound and dynamic images (Al-Qaysi et al., 2020). This way not only directly arouses people’s auditory resonance, but also satisfies people’s visual aesthetic feeling. Compared with paper media, it can make people get information in the shortest time (Oakley et al., 2017). It can be said that the film and television media works are a new art form which combines literature, music, painting, photography, and other arts. It organically integrates the characteristics of other art forms, and exerts this characteristic vividly through moving images (Ramasubramanian, 2016).

#### Orientation

It is precisely because of the directional characteristics of the film and television media that the aesthetics of the viewers or contemporary college students will be more affected (Martin and Frenette, 2017). The correct guidance will produce good social education function and cultural propaganda function, enrich people’s inner world, inspire people’s soul, and promote people’s growth, but wrong guidance will inevitably have negative impacts. In this regard, through the design of questionnaires, the impact of film and television media on college Students’ cultural art aesthetic in the new media era is analyzed.

Given the above analysis, in addition to the characteristics of two-way interaction and multiple selection, the characteristics of film and television media also include immediacy, popularization, intuition and guidance. Through the above analysis of the characteristics of film and television media, it can be seen that film and television works of art are inevitable for college Students’ aesthetic perception ability. Therefore, how to strengthen the combination of film and television media and college Students’ aesthetic culture education is a problem needs to be solved at present. In view of this, through questionnaires, this paper draws two opinions and methods on the reform of the current aesthetic culture education in China.

On the one hand, the course of film and television media appreciation should be added, which should be a compulsory course. In the new media era, film and television works are uneven. Choosing the right film and television works will help to improve the aesthetic interest of college students. College students are at the critical stage of physiological and psychological development, and good guidance will certainly promote the formation of their healthy personality. Therefore, it is suggested that colleges and universities not only offer courses of film and television media appreciation, but also need to change such courses from elective to compulsory, so that each college student can learn to distinguish high-quality film and television works under the correct guidance of teachers. In this way, students can learn certain correct aesthetic interest and values in the process of leisure and entertainment, and then cultivate their sound personality.

On the other hand, teachers need to strengthen their own reserves of film and television art. Teachers, as the pioneers of “preaching, receiving and dispelling professional doubts,” must have a certain self-view of film and television media works. Teachers’ profound aesthetic culture and film and television culture reserves are the prerequisite for teachers to form their own unique views on film and television media art and to excavate aesthetic highlights in teaching. Only in this way can they provide correct guidance for Students’ aesthetic appreciation. At the same time, teachers should attach importance to the use of their own emotional understanding and Students’ emotional communication and guidance of aesthetic culture education in the process of aesthetic culture education, so that students can have certain emotional resonance and truly understand the aesthetic interest of film and television art.

### The Influence of Film and Television Culture on College Students’ Values

Values refer to the general views of the subject on the value of the objective object, that is, people’s understanding of the satisfied and being satisfied relationship between the subject and object or the meaning presented to the subject. It is a systematic and relatively stable value choice structure system composed of value objectives, value orientation and value evaluation. Values are contemporary college Students’ subjective understanding of life direction and value significance based on self-cognition, which can well guide their ideology and behavior. Contemporary college students are in a critical period of the formation and establishment of values. In social activities, their values will also change with the influence of living environment, mainstream values and socialist ideological trend. As a crucial part of contemporary college Students’ cultural life, film and television culture is superior to other educational media in intuitive visibility, artistic aesthetics and value orientation. It exerts an unprecedented and important impact on the knowledge structure, ideas, behavior, ideology, and values of contemporary college students.

(1) Film and television culture and contemporary college Students’ political values. Political values are “the basic yardstick for political people to judge political things,” including political party, state, nation, politics, law, and belief. It is mainly reflected in the recognition of the party’s decision-making, the understanding of the party’s basic line, the observance of the political system, whether the political attitude is clear and whether the political enthusiasm is high. Political values are the most basic identification of contemporary college students with national policies and national emotions, and the political guarantee to improve cultural self-confidence. The establishment of correct political values by contemporary college students is conducive to their correct understanding and scientific political evaluation of political phenomena and political behaviors in social life (Yang, 2020). Film and television culture has a unique value output function. Film and television works with strong political value orientation are a challenge to the political values of contemporary college students. The “political” factors in the creation of film and television cultural works are embodied in the class view, the bottom perspective and the perspective of social analysis. Through the influence strategy and narrative skills of the film, the understanding of modernity problems such as city, class, unemployment, money, and enlightenment is rewritten in various skills and ways. Film and television culture has both positive and negative effects on contemporary college Students’ political values.

(2) Film and television culture and contemporary college Students’ moral values. Moral values and literary works go hand in hand. An excellent literary work must show its distinct moral value orientation in different ways, and has the function of thought-provoking moral education. With the specific and perceptible screen reproduction technology, film and television culture has become one of the most expressive, infectious and influential modern literary and artistic forms among multiple literary and artistic forms. The moral values of contemporary college students determine their ethical concepts, behavior concepts and moral evaluation standards. Whether the moral values of contemporary college students are scientifically correct or not greatly affects the contemporary college students to establish lofty moral ideals. The influence of film and television culture on the moral values of contemporary college students should be analyzed from a dialectical perspective. Excellent film and television cultural works can bring moral and emotional enlightenment to contemporary college students and purify their hearts. For example, the kindness, purity and selflessness of the protagonist in the 1990s TV drama *Desire* fully show the virtues of the traditional oriental society. It is in sharp contrast to the “money worship” and “money first” in the social environment at that time, and has the function of moral education and cultivating human nature.

(3) Film and television culture and contemporary college Students’ professional values. Professional values condense the judgment and evaluation of contemporary college Students’ personal ability, behavior and attitude during job selection. It also includes the judgment criteria of contemporary college Students’ professional nature and types. Professional values have a strong goal and orientation, directly affecting contemporary college Students’ attitude and way of choosing a job. It determines the professional nature of contemporary college students after completing the internship of higher education, and is related to the specific content of their social practice activities and the realization of personal and social values (Martin and Schroeder, 2021). During socialist market globalization, film and television works show the characteristics of a wide variety, huge quantity, and uneven quality. Excellent film and television cultural works exert a positive and healthy impact on the values of contemporary college students. For example, the film *Not One Less* shows the excellent quality of a teacher’s love and dedication for the occupation, and forms a good demonstration for contemporary college students to shape their professional values. On the contrary, if film and television works deviate from the creative principle of “truth, goodness and beauty,” it will have a negative impact.

(4) Film and television culture and contemporary college Students’ aesthetic values. Marx and Engels proposed the aesthetic propositions and concept in the organic unity of history and aesthetics. Marx’s “labor creates beauty,” “man also builds according to the law of beauty,” and “beauty is the objectification of man’s essential power,” as well as Engels’s “truly reproduce the typical characters in the typical environment” all refer to the practice of artistic production and aesthetic practice. (A). Although Marx and Engels did not directly point out the historical inevitability of aesthetic practice, they affirmed that aesthetics came into being with practice, and also reflected the subjective initiative of the Subject’s aesthetic consciousness and aesthetic value of social production practice. Aesthetic consciousness is the conscious activities of aesthetic perception, aesthetic experience, aesthetic creation, and aesthetic appreciation in artistic activities. (B). The emergence of aesthetic consciousness reflects the historical inevitability of the emergence of aesthetic values. Aesthetic values are the aesthetic standard of aesthetic consciousness, and the presentation of aesthetic consciousness. It mainly includes personal aesthetic standards and personal understanding of spiritual beauty, appearance beauty, content beauty and form beauty. (C). Writers’ creation needs aesthetic consciousness and aesthetic ability. Similarly, people generally have aesthetic ability for literary works in life, but the aesthetic ability, aesthetic angle, aesthetic standard, and aesthetic evaluation methods are different. People’s behavior and thinking mode in practical activities determine aesthetic values, which have a reaction to people’s spiritual world, ideological beliefs, behavior activities, and value emotions (Wang et al., 2021). As the main body of higher education, contemporary college Students’ aesthetic ability, and aesthetic value orientation determine the social aesthetic standard. Film and television culture transmits the “artistic beauty” of film and television works of art through vivid videos and pictures, which is also the “beauty of life” and “beauty of science and technology.” Film and television culture uses lens art to create vivid screen characters. These characters can cultivate and educate people, guide contemporary college students to establish correct aesthetic values, distinguish the “truth, goodness, and beauty” in life in social production practice, inspire the wisdom of discovering beauty, and cultivate the ability to recognize the beauty of nature, life and harmony, so as to stimulate the spiritual power and promote the all-round personal development of contemporary college students.

### Feasibility Analysis of Art Education Promoting College Students’ Entrepreneurship Education

(1) The goal of art education and entrepreneurship education for college students is unified.

By popularizing the basic knowledge of art, college Students’ art education improves their artistic cultivation to improve their aesthetic psychological structure and open and improve their perception, imagination, and creativity. It can cultivate a perfect personality and promote the harmonious development of college students. College Students’ art education is important content and an effective way to implement aesthetic education and quality education in colleges. It is an important carrier and effective channel to comprehensively improve college Students’ cultural literacy and comprehensive quality. The core goal of college Students’ art education is to cultivate college students with all-round development. Entrepreneurship education refers to the flexible use of educational technology, the integration and optimization of educational resources, the organic integration of discipline advantages, and the effective expansion of educational channels. It can help college students cultivate entrepreneurial awareness, shape entrepreneurial personality, enrich entrepreneurial knowledge and improve entrepreneurial ability. College Students’ entrepreneurship education is an important part of quality education. It is the main channel and an important way to expand college Students’ entrepreneurial skills, comprehensive quality, and professional skills. Its core content is to cultivate and improve the entrepreneurial quality of college students and promote their all-round development. To sum up, it reveals that college Students’ art education and entrepreneurship education are actually an important part of college Students’ quality education and innovation education. It is an effective expansion of the function of higher education, which aims to improve the comprehensive quality of college students and realize people-oriented scientific development (Wu et al., 2021).

(2) The timing of college Students’ art education is consistent with that of their entrepreneurship education.

In the twenty-first century, human society has entered the era of the knowledge economy. The competition of comprehensive national strength among countries depends on the competition of talents. The knowledge economy puts forward unprecedented and urgent requirements for talent training, emphasizing that educators must cultivate Students’ comprehensive ability and good comprehensive quality. College students are spokesmen and entrepreneurs in the era of the knowledge economy. To better participate in international competition in the era of the knowledge economy and the tide of the historical process of global economic integration, China must cultivate and have more fully developed entrepreneurial talents with good comprehensive quality. Strengthening college Students’ art education and entrepreneurship education is a necessary measure to comprehensively promote quality education, cultivate and bring up a new generation of high-quality talents in the twenty-first century and enhance China’s comprehensive national strength. The party and government attach great importance to college Students’ art education and entrepreneurship education, and provide a good policy environment for it. Higher education should cultivate college Students’ innovative ability, practical ability and entrepreneurial spirit, and generally improve college Students’ humanistic and scientific literacy. Art education and entrepreneurship education of college students help to improve the ideological and moral quality and cultural cultivation of college students, and promote the intellectual development and creativity of college students. It is beneficial to college Students’ mental health and personality improvement, and is conducive to the overall improvement of college Students’ entrepreneurial quality. The proposal and promotion of the two are the inevitable choices for the development of the era of the knowledge economy. It is consistent with the inherent requirements and timing of China’s high education reform and development.

### The Value of College Students’ Cultural and Aesthetic Education Under the Background of Innovation and Entrepreneurship Education

At present, there is still a lack of aesthetic and creative courses integrating theoretical appreciation, aesthetic practice and innovation in innovation and entrepreneurship courses in colleges. In aesthetic education, the consciousness of cultivating students to discover, understand and practice beauty is not high, and there is no deep fit between beauty and creation. The consciousness of relying on large-scale artistic creation and artistic performance to cultivate Students’ ability of innovation and creation is not high. The aesthetic emotion and aesthetic feeling of students in innovation and entrepreneurship are not comprehensively realized. Aesthetic education often focuses on the “activity” level, which has not yet risen to the “education” level, and is not close enough to the “gene” education goal buried in innovation and entrepreneurship education. Although many college students pursue spiritual height, the aesthetic distance between elegant art culture and culture accepted by the public is large. College culture is at the forefront of social and cultural development. It requires college to focus on the aesthetic cultural phenomena in the current society, and explore the aesthetic connotation hidden behind those specific phenomena. The popular culture represented by commodity culture should become the object of resistance and elimination of the main freedom of human spiritual life and the diversity of civilization development in ideology while prospering on campus. Education proposes that cultural consumption should improve the cultural quality of workers and finally mobilize the enthusiasm of the public, which has become a practice of socialist significance, that is, the purpose of cultural consumption is still a link of production. Therefore, young people need to forge their own internal transcendence pursuit in the atmosphere of mass culture.

Although art education is an important part and way to implement cultural aesthetic education, it is not the whole content and form of education. Perceptual education enriches people’s emotions. Through aesthetic art activities, people’s senses are more sensitive and vivid, so as to continuously stimulate individual emotion, imagination and creativity. Literature plays a special role in the detachment of life and the pleasant cultivation of temperament. It advocates the unity of truth, goodness and beauty and knowledge, emotion and meaning. In addition to physical fitness, the psychological needs of truth, goodness and beauty must be met. Aesthetic education emphasizes creativity. In addition to the special creative ability, it emphasizes more about its most basic connotation, which is the individual’s continuous realization of renewed life vitality. It is the basic characteristic and ability of healthy individual life, as well as the foundation and source of special creative ability.

Aesthetics and art are the best forms of education to develop and cultivate college Students’ creativity. Especially, China vigorously advocates mass entrepreneurship at present. Under the background of mass innovation and innovation driven era, the theoretical research and practice of aesthetic education should learn from the experience of many developed countries, and take promoting the development of individual creativity as the basic task of aesthetic education and art education. Creative performances are based on the combination of aesthetic education theory and practice. Through creative aesthetic activities, individuals show strong imagination and practical ability. Especially, they are more focused and engaged, and pay attention to the beauty and pleasure in the process. In the post epidemic era, the offline teaching mode of entrepreneurship and innovation education has been strongly impacted, and offline entrepreneurship and innovation education is difficult to carry out in essence, which is the dilemma faced by entrepreneurship and innovation education. Meanwhile, the normalization of online teaching mode and online and offline mixed teaching mode has made entrepreneurship and innovation education resolve the crisis. During the epidemic period, the online teaching mode made a substantial breakthrough, especially for the effect of entrepreneurship and innovation education. Online lectures and live classes break the restrictions of time and space and can be shared by multiple schools and even the whole country. Such online and offline mixed mode of entrepreneurship and innovation education can realize the communication and interaction within the school, and let teachers and students understand the national situation and realize global interaction.

### Questionnaire Survey

The above strategies are based on the investigation and analysis of this questionnaire, so the specific investigation and analysis will be focused on. Survey questions are designed. College students from five colleges in Xi’an are taken as the research subjects to explore the specific impact of film and television media on college Students’ cultural aesthetics in the new media era. The questionnaire is anonymous. Overall, 250 questionnaires are distributed (50 questionnaires in each school), and 250 are recovered. After some invalid questionnaires such as incomplete filling are deleted, 235 valid questionnaires are obtained, with an effective rate of 94%. There are six relevant research questions, and the specific contents are shown in Table 1.

**TABLE 1:** List of research content.

Questions	Concrete content
1	In what ways do you increase your aesthetic interest in your daily life?
2	What is your favorite campus cultural activities?
3	Do you shoot a barrage during the movie?
4	Average daily time spent watching movies and TV?
5	Will the things that movie stars used stimulate your purchasing desire?
6	Will you take the course of film and television media appreciation offered by the school?
7	How often do you forward idol information on social platforms?

The actual effect of college aesthetic education is mainly reflected in the aesthetic quality of college students, which is closely related to their cognition of the value of aesthetic education and the promotion of college aesthetic education. The self-evaluation of college Students’ aesthetic literacy and aesthetic education value cognition and the evaluation and expectation of school aesthetic education are investigated. It is hoped that the supply-side reform of college aesthetic education can be promoted from the research and analysis of college Students’ demand side. Teachers in ideological and political education and sociology majors modify and delete inappropriate, repetitive and research-irrelevant topics to ensure the scientificity and operability of the questionnaire.

## Results and Discussion

### Specific Situation of College Students’ Cultural Aesthetics

**FIGURE 1:**
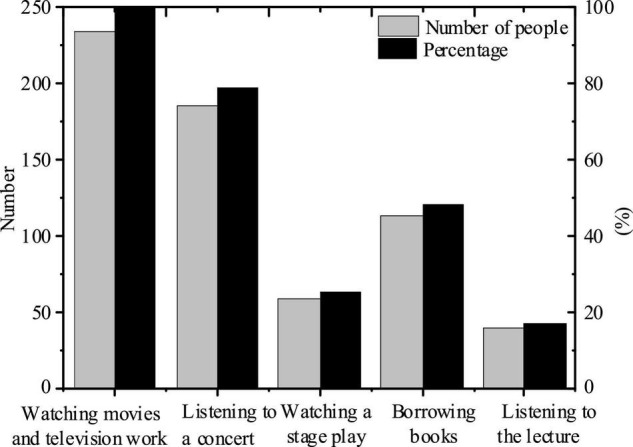
The proportion of various ways for college students to improve their self-aesthetic ability in daily life.

For this part of the analysis, one-one analysis is carried out in accordance with the above-mentioned settings. For the answer design in question 1, five items for multiple choices are designed, namely, borrowing books; attending lectures; watching movies and television works; listening to a concert and others, specifically, as shown in Table 2 and Figure 1.

**TABLE 2:** Ways to improve college Students’ self-aesthetic ability in daily life.

Choices	Watching movies and television works	Listening to a concert	Watching a stage play	Borrowing books	Attending lectures
Number of people	235	185	59	113	40
Percentage	100%	78.7%	25.1%	48.1%	17.0%
					

Through the above analysis, it can be found that the main way to improve college Students’ aesthetic ability is to watch movies and TV works, while the lowest proportion is to listen to lectures to improve their self-aesthetic ability, which is 17.1%. It can be seen that movies and TV works of art have the greatest impact on the university’s aesthetic ability, and they should also arouse our attention.

**FIGURE 2:**
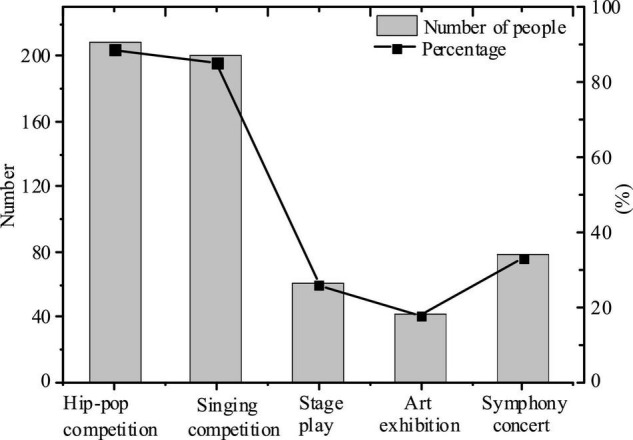
The proportion of college Students’ favorite campus cultural activities.

For Question 2, four options are set for a number of choices, namely hip-hop competition, singing competition, stage play, art exhibition, and symphony concert, as shown in Figure 2 and Table 3.

**TABLE 3:** College Students’ favorite campus cultural activities.

Choices	Hip-hop competition	Singing competition	Stage play	Art exhibition	Symphony concert
Number of people	209	201	61	42	78
Percentage	88.9%	85.5%	26.0%	17.9%	33.2%

From the table and figure, it is seen that college students prefer hip-hop competitions and singing competitions more, which is closely related to the singing and dance elective programs in the popular film and television variety arts in recent years. On the contrary, they are less interested in stage plays, symphony concerts, and art exhibitions with higher aesthetic requirements. It can be seen that under the influence of contemporary film and television media, college Students’ aesthetic ability tends to be visualized.

For Question 3, single choices are set. The specific data and statistics are shown in Table 4.

**TABLE 4:** Statistical analysis of data of sending barrage of college students in the process of watching films.

Choices	Yes	No
Number of people	198	37
Percentage	84.3%	15.6%

Through investigating the two-way interaction of film and television media in the context of new media, it is found that the proportion of sending barrage of contemporary college students in watching film and television works is much higher than that of not sending barrage. It can be seen that contemporary college students are more willing to express their own views and aesthetic attitudes toward film and television works, and it also shows that college students are more affected by the complaint-making through the “culture” of barrage. However, the real-time bullet screen also negatively impacts the viewing experience, such as episode-related, distraction, being influenced, and some sequelae.

For Question 4, four options are set for single choice, which are 1–3, 3–5, 5–7, and more than 7 h, as shown in Table 5.

**TABLE 5:** The average daily time spent by college students in watching movies and TV works.

Choices	1–3 h	3–5 h	5–7 h	More than 7 h
Number of people	40	162	23	10
Percentage	17.0%	68.9%	9.8%	4.3%

By comparing the time consumed by college students in watching movies and TV programs, it is known that more than half of the students spend 3–5 hours a day watching movies and TV works, and the proportion of more than 7 h is the lowest. This also proves that the art works of movies and TV works have become a part of college Students’ daily life. It suggests that contemporary college students watch film and television works every day, but the viewing time is different.

For Question 5 and Question 6, they are single choice, as shown in Table 6.

**TABLE 6:** The influence of stars in film and television on college Students’ consumption and the statistics of their preferences for setting film and television art appreciation courses.

Choices	Yes	No	Choices	Willing	Unwilling
Number of people	210	25	Number of people	165	70
Percentage	89.4%	10.6%	Percentage	70.2%	29.8%

According to the influence of advertisement implantation on college Students’ consumption, 89.4% of students said they would be affected by it. This is the reason why there are more and more advertisement implanted in film and television works in recent years. At the same time, based on the preference for offering film and television appreciation courses in colleges and universities, there are still many students willing to listen to such courses, which shows that it is very wise to incorporate film and television appreciation courses into the teaching system.

According to the above research, the author will specifically analyze the influence of film and television media on college Students’ aesthetics in the context of new media in the following.

### Cultural and Artistic Aesthetic Education of College Students

Table 7 shows that these five schools’ cultural and artistic aesthetic education courses are mainly music courses and dance courses. Music courses are the most, followed by dance courses. In addition, the cultural and artistic aesthetic education courses to be added in these five colleges are mainly music courses and dance courses, which shows that the courses offered cannot meet the needs of students. In addition, college students have a great demand for film and television courses.

**TABLE 7:** Cultural and artistic aesthetic education courses.

Choices	Music	Fine art	Dance	Drama	Film and television	Poetry and literature	Other courses
Number of people	162	76	89	40	61	20	13
Percentage	68.9%	32.3%	37.9%	17.0%	26.0%	8.5%	5.5%

In the survey of college Students’ demand for cultural and artistic aesthetic education courses (Table 8), college students put the improvement of relevant courses in the first place, which fully reflects college Students’ ardent expectation for the improvement of cultural and artistic aesthetic education courses. Generally, colleges need to improve the supervision and evaluation system, the credit-related graduation mechanism and the introduction of social resources.

**TABLE 8:** College Students’ demand for cultural and artistic aesthetic education courses.

Choices	Improve the curriculum	Improve the system of practical activities	Improve the supervision and evaluation system	Credit-related graduation mechanism	Introduction of social resources
Number of people	145	122	112	83	62
Percentage	61.7%	51.9%	47.7%	35.3%	26.4%

## Discussion

Through the above research, the impact is summarized as two levels of positive impact and negative impact for analysis.

The positive impact is reflected as the film and television media providing a good way for the cultivation of contemporary college Students’ aesthetic perception ability. With the development of new media technology, film and television media works gradually show strong appeal and perfect special effects. Its plastic arts, spatial forms, special effects, and the dynamic and static artistic conception of the picture will provide some reference for contemporary college Students’ aesthetic perception ability. It is also because of the comprehensive performance of film and television media art and the characteristics of multi-way integration that music, literature and drama can be organically combined in space, sculpture, painting, and architecture can be integrated in time. While college students can experience their legendary and profound stories in their grand scenes, beautiful music and dialogues, and then get emotional instinct resonance to achieve psychological and spiritual satisfaction, so as to improve the aesthetic level and promote the development of aesthetic perception ability.

In addition, the positive influence of film and television media on college students is also reflected in the correct establishment of college Students’ aesthetic values. The rich artistic beauty in film and television art is also conducive to cultivating college Students’ sentiment, such as Japanese animation, Korean dramas in South Korea, American dramas, and films in the United States. Through watching films and TV works, college students are influenced by the beauty of nature, form, content and humanistic artistic conception in film and TV art, thus improving their artistic appreciation and creativity. The presentation of these artistic beauty will purify the inner world of college students, beautify their spiritual realm and develop toward the good, and consciously standardize themselves with the standards and behaviors of beauty, so as to achieve the unity of truth, goodness, and beauty.

Negative effects are mainly reflected in two levels, namely, the vulgarization of film and television media works and the consumption of aesthetic ideas. With the development of modern society and economy, the film and television media has become the companion of market economy. Its commercialized characteristics determine that the film and television art must rely on the market, and then use the vulgar way to please the public for profit. It is embodied in showing the real scene through flashy or unrealistic life style that college students yearn for. But such scenes are difficult to achieve in real life, which will make college students intoxicated with this, confuse the gap between realistic and unrealistic scenes, and then excessively pursue unrealistic scenes and lose themselves. Through this questionnaire survey, it can be concluded that the singing competition and hip-hop competition rank the top two in the campus activities that college students like. Compared with more elegant and artistic symphony concerts, stage plays and exhibitions of works of art, they have received little attention from college students. This biased aesthetic feeling is very unfavorable for college students to establish aesthetic standards of art, which not only makes them unable to appreciate the beauty correctly, but also affects their healthy and upward aesthetics, and destroys the healthy learning atmosphere in university campuses.

Colleges should fully realize the importance of cultural and artistic aesthetic education to promote the all-round development of college students, and earnestly implement the spirit of relevant national documents. Students’ evaluation reflects their own feelings about cultural and artistic aesthetic education, which is not too high. The reasons may be Students’ attention and the degree of school publicity and implementation. It reveals that colleges should further strengthen the publicity and guidance of cultural and artistic aesthetic education, improve the related teaching and carry out aesthetic and practical activities to allow the majority of students to participate.

The consumerism of aesthetic concept is mainly embodied as consumption has become a part of people’s daily life in today’s era. It is undeniable that consumption not only meets people’s material and psychological needs, but also brings disadvantages. In order to better promote the sales of sponsored products, film and television works often incorporate some advertisements, such as the phenomenon of advertising theatre in popular film and television works in recent years, which makes the spiritual production commercialized and art purely become a consumer product. For contemporary college students, the use of excessively gorgeous costumes and luxury goods in movies and television makes them gradually adapt to this consumption concept and consumption mode, and gradually develop the habit of highlighting and showing off their personality through consumption in aesthetics to be different from other social groups. For example, at this stage, the rise of “Hanfu” culture among college students makes too many college students regard this kind of consumption as a symbol of higher quality of life and a symbol of happy life, which leads to the prevalence of hedonic consumption behavior and promotes the evil habit of conspicuous consumption. Besides, various online platforms are full of short videos. This kind of entertainment is a waste of time and will affect college Students’ learning. Therefore, it is necessary to improve college Students’ aesthetic ability.

## Conclusion

Film and television culture and art have a positive and negative impact on college Students’ cultural aesthetic perception. The positive influence is that film and television media provides a good way for the cultivation of contemporary college Students’ aesthetic perception ability, and also helps to establish college Students’ aesthetic values correctly. The negative impact is mainly reflected in two levels, namely, the vulgarization of film and television media works and the consumption of aesthetic concepts. How to guide college Students’ cultural aesthetic education correctly, there are two suggestions. It is suggested that the course of appreciation of film and television media works of art should be taken as a compulsory course. Meanwhile, teachers need to strengthen their reserves for film and television works to better guide college students to distinguish between good and bad film and television works and help them obtain more emotional improvement, get improvement in artistic aesthetic ability, and establish a sound personality in the process of leisure and entertainment. In this way, students will not waste their time and life through film and television works, or even learn the wrong aesthetic interest in bad film and television works, which will affect the formation of correct values. Meanwhile, the school should strengthen aesthetic education to help college students inspire people to think about their own existence value and life significance through aesthetic education. For example, Japan has animation, South Korea has Korean dramas, and the United States has American dramas and films. It can be said that nowadays, film and television are full of people’s daily life, coupled with the development of the Internet and the popularity of mobile devices. Therefore, helping college students distinguish the film and television works of art will become an important part of college Students’ aesthetic ability education and even personality training in the future. With the continuous development and progress of the times, the advantages and educational role of film and television media art have attracted extensive attention of worldwide educators. Moreover, many colleges have set up corresponding film and television media courses and implemented modern education in film and television communication, which also makes the advantages of film and television media art more prominent. Therefore, the study of film and television media has important practical significance for improving college Students’ aesthetic value and the formation of personal moral quality and cultivation.

The research deficiency is that the number of samples is relatively small, and the results are limited. In the later stage, the sample size will be increased to improve the universality of the results.

## Data Availability Statement

The raw data supporting the conclusions of this article will be made available by the authors, without undue reservation.

## Ethics Statement

The studies involving human participants were reviewed and approved by the Zhijiang College of Zhejiang University of Technology Ethics Committee. The patients/participants provided their written informed consent to participate in this study. Written informed consent was obtained from the individual(s) for the publication of any potentially identifiable images or data included in this article.

## Author Contributions

All authors listed have made a substantial, direct, and intellectual contribution to the work, and approved it for publication.

## Conflict of Interest

The authors declare that the research was conducted in the absence of any commercial or financial relationships that could be construed as a potential conflict of interest.

## Publisher’s Note

All claims expressed in this article are solely those of the authors and do not necessarily represent those of their affiliated organizations, or those of the publisher, the editors and the reviewers. Any product that may be evaluated in this article, or claim that may be made by its manufacturer, is not guaranteed or endorsed by the publisher.
